# Interactions among variants in P53 apoptotic pathway genes are associated with neurologic deterioration and functional outcome after acute ischemic stroke

**DOI:** 10.1002/brb3.1492

**Published:** 2020-01-07

**Authors:** Xingyang Yi, Qiang Zhou, Guo Sui, Gaoping Ren, Lili Tan, Jie Li, Jing Lin, Shaozhi Bao

**Affiliations:** ^1^ Department of Neurology The People's Hospital of Deyang City Deyang Sichuan China; ^2^ Department of Neurology The Third Affiliated Hospital of Wenzhou Medical University Wenzhou Zhejiang China; ^3^ Nursing Department People's Hospital of Deyang City Deyang Sichuan China

**Keywords:** genetic polymorphism, ischemic stroke, *MDM‐2*, *MMP‐9*, neurologic deterioration, outcome, *P53*

## Abstract

**Objective:**

Neurologic deterioration (ND) and functional outcome after ischemic stroke (IS) are not accurately predicted by clinical pictures on admission. The aim of present study was to investigate the association of variants in P53 apoptotic pathway genes with ND and functional outcome after IS.

**Methods:**

Genotypes of nine variants in apoptosis‐relevant genes were measured in patients with acute IS. Gene–gene interactions were analyzed by generalized multifactor dimensionality reduction (GMDR). The primary outcome was ND. ND was diagnosed in patients who worsened ≥2 points (National Institutes of Health Stroke Scale [NIHSS] score) within the first 10 days of stroke onset. The secondary outcome was functional status at 90 days after IS as measured by modified Rankin Scale (mRS) score.

**Results:**

A total of 705 enrolled patients, ND occurred in 174 (24.7%) patients, and 184 (26.1%) patients were poor functional outcome (mRS score > 2). Although the nine variants were not significantly associated with ND and functional outcome by univariate analysis, there was a gene–gene interaction among *P53*rs1042522, *MDM‐2*rs2279744, and *MMP‐9* rs3918242 using GMDR analysis. The high‐risk interaction among the three variants was independently associated with higher risk of ND (HR, 2.04, 95% CI: 1.22–5.64, *p* = .018) and poor functional outcome (OR, 2.68, 95% CI: 1.68–7.86, *p* = .004) after adjusting for the covariates.

**Conclusion:**

The interactions among *P53* rs1042522, *MDM‐2* rs2279744, and *MMP‐9* rs3918242 may increase the risk of ND and poor functional outcome and may be considered as a genetic marker of predicting ND and poor functional outcome after stroke.

## Graphical abstract

(a) The neurologic deterioration (ND) and functional outcome after ischemic stroke (IS) are not accurately predicted by clinical pictures on admission. (b) There was a gene–gene interaction among *P53* rs1042522, *MDM-2* rs2279744, and *MMP-9* rs3918242 using GMDR analysis. (c) The interactions among *P53* rs1042522, *MDM-2* rs2279744, and *MMP-9* rs3918242 may increase the risk of ND and poor functional outcome and may be considered as a genetic marker predicting ND and poor functional outcome after stroke.

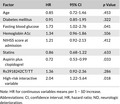

## 1 BACKGROUND

Stroke is the first cause of death and adult disability in China (Guan et al., 2017), with approximately 80% being ischemic strokes (IS). Neurologic deterioration (ND) occurs in one third of patients with acute IS, and it is a devastating complication and associated with increased mortality and long‐term functional disability (Vahidy et al., 2014; Yi, Han, Zhou, Lin, & Liu, 2016). However, ND or functional outcome after IS remains largely unpredictable (Weimar, Ziegler, König, & Diener, 2002). Patients with initially a similar clinical picture can worsen or improve dramatically within the first days after IS (Banks & Marotta, 2007; Castillo, 1999). Neuronal apoptosis in the ischemic penumbra may be an important mechanism for ND and impaired functional recovery of IS patients (Broughton, Reutens, & Sobey, 2009; Sairanen, Karjalainen‐Lindsberg, Paetau, Ijäs, & Lindsberg, 2006). Thus, the variable prediction of ND or functional outcome after stroke could be the effect of different genetic backgrounds to apoptosis.

Apoptosis is also called programmed cell death, and it is an important mechanism of delayed ischemic brain damage in animal experiments (Sairanen et al., 2006). Two general pathways of apoptosis are triggered after cerebral ischemia, that is, intrinsic pathway (originating from mitochondrial release of cytochrome c) and the extrinsic pathway (originating from the activation of cell surface death receptors) (Broughton et al., 2009). Genetic polymorphisms of cell‐cycle regulating genes may affect the DNA damage (Yagnik, Jahangiri, Chen, Wagner, & Aghi, 2017). When cells are damaged, cell division will stop in G1 phase of mitosis (Duan et al., 2017). In the cell‐cycle regulation, the arrest of cell cycle in G1/S transform depends on p53 process (Yousefi, Rahmati, & Ahmadi, 2014), which is the master control system of the cell cycle, cell apoptosis, and genome stability. *P53* encodes p53 transcription factor, a tumor suppressor protein that can mediate apoptosis in eukaryotic cells. Recent studies have shown *P53 Arg72Pro* (rs1042522) polymorphism triggers neuronal death via the mitochondrial apoptotic pathway (Gomez‐Sanchez et al., 2011). *P21*, the downstream gene of the *P53* gene, can prevent cell‐cycle progression in the G1/S and G2/M phases and plays a key role in suppressing cancer (Karimian, Ahmadi, & Yousefi, 2016). The murine double minute 2 (*MDM‐2*) gene is a negative regulator of *P53*, which can inhibit *P53* expression (Moumen, Patane, Porras, Dono, & Maina, 2007), and *P53* may upregulate *P21* expression in response to DNA damage (Macleod et al., 1995). Rs1042522 polymorphism may affect neuronal vulnerability to apoptosis and can be considered as a genetic marker for poor functional outcome after stroke (Gomez‐Sanchez et al., 2011). Many studies have shown that single nucleotide polymorphisms (SNPs) of *P53*, *MDM‐2*, and *P21* rs1801270 play important roles in DNA damage and apoptosis and are intimately related to cancer occurrence (Chen et al., 2015; Duan et al., 2018). Matrix metalloproteinase‐9 (MMP‐9) can activate numerous pro‐inflammatory cytokines and chemokines such as interleukin and tumor necrosis factor, facilitates leukocytes transport across the endothelium, and plays a key role in neuronal damage, apoptosis, and blood–brain barrier (BBB) destruction after cerebral ischemia (Barr et al., 2010; Candelario‐Jalil, Yang, & Rosenberg, 2009). Polymorphisms of *MMP‐9* gene regulate the transcription of MMP‐9 protein and are associated with increased IS or cancer risk (Yuan et al., 2013; Zhu, Liu, Zhou, & Chen, 2018). So far, no study has reported the correlations between the SNPs of *P53*, *P21*, *MDM‐2*, and *MMP‐9* genes and ND or functional outcome after IS.

Neurologic deterioration and functional outcome after IS may be very complex, and a single polymorphism in a particular gene is unlikely to explain completely the complex genetic etiology for ND and functional outcome. Gene–gene interactions or gene–environmental interactions may synergistically contribute to ND and functional outcome (Yi, Liao, Fu, Zhang, & Wang, 2015). Generalized multifactor dimensionality reduction (GMDR) analysis is customarily used to assess the higher order gene–gene or gene–environment interactions (Lou et al., 2007). However, the potential effects of gene–gene interactions in apoptotic ‐relevant genes on ND and functional outcome after IS are unclear. In this study, therefore, we aimed to investigate the association of gene–gene interactions among apoptotic‐relevant genes with ND and functional outcome after IS, which could provide more insights into the genetic background for ND and functional outcome, prevent ND for better, and improve functional outcome.

## 2 METHODS

### 2.1 Ethics statement

This study was conducted in the People's Hospital of Deyang City and the Third Affiliated Hospital of Wenzhou Medical University between March 2014 and December 2016. The study protocol was approved by the Ethics Committee at the participating hospitals in accordance with the principles stated in the Declaration of Helsinki. Written informed consent was obtained from each of the participants before participating in the study.

### 2.2 Study population

Between March 2014 and December 2016, we consecutively registered patients who had suffered their first‐ever IS and were admitted to the participating hospitals within the first 48 hr after onset of symptoms. IS were confirmed on the basis of both clinical findings and brain magnetic resonance imaging (MRI) scan. All patients underwent computed tomography or MR angiography of the brain, carotid duplex ultrasound, common electrocardiogram (ECG), or 24‐hr Holter ECG, as well as echocardiogram. The inclusion criteria were as follows: (a) age ≥ 40 years old and (b) National Institutes of Health Stroke Scale (NIHSS) score ≤ 15 points on admission. Exclusion criteria were as follows: (a) thrombolytic therapy or thrombectomy; (b) NIHSS score > 15 points at admission; (c) severe cardiovascular, liver, and renal disease; (d) other determined etiology or undetermined etiology stroke according to new subtype classification criteria (Han et al., 2007); (e) hypoxia, fever, or any relevant hemodynamic compromise on admission; and (f) unwilling to participate in this study. All enrolled patients received standard therapy according to the guidelines (Jauch et al., 2013; Kernan et al., 2014).

### 2.3 Clinical variables

Medical history and vascular risk factors were recorded on admission. Fasting blood samples from patients were assessed for glucose, triglycerides (TG), total plasma cholesterol (TC), and low‐density lipoprotein cholesterol (LDL‐C). Hyperlipidemia was defined as TG > 180 mg/dl, TC > 200 mg/dl, or use of lipid‐lowering medication (Yi et al., 2016). Stroke subtypes were classified according to the new subtype classification criteria (Han et al., 2007). Stroke severity was evaluated by a certified member of stroke team using the NIHSS on admission.

### 2.4 Outcome variables

For each patient, NIHSS was assessed by a member of stroke team on admission and subsequently daily during the period of hospitalization. Functional outcome was evaluated at 3 months using the modified Rankin Scale (mRS). The primary outcome was ND. ND was diagnosed in patients who worsened ≥2 points (NIHSS) within the first 10 days of stroke onset after excluding a new infarct in another vascular territory or hemorrhagic transformation (HT) (Yi et al., 2016). The secondary outcome was functional status at 90 days after IS. mRS score > 2 was considered as poor functional outcome, and mRS score ≤ 2 was defined as good functional outcome (Swieten, Koudstaal, Visser, Schouten, & Gijn, 1988).

### 2.5 The selection of SNPs and genotyping

In this study, SNPs of apoptotic‐relevant genes were selected from the NCBI database (http://www.ncbi.nlm.nih.gov/SNP), according to the following criteria: (a) These SNPs have been evaluated in previous studies (Gomez‐Sanchez et al., 2011; Moumen et al., 2007; Yuan et al., 2013; Zhu et al., 2018) and (b) minor allele frequency for these SNPs > 0.05. According to the criteria, nine variants were assessed, including *P53* rs1042522, *MDM‐2* rs2279744, *MDM‐2* rs1690916, *P21* rs1801270, *MMP‐9* rs1056628, *MMP‐9* rs3918242, *MMP‐9* rs17576, *MMP‐9* rs3787268, and *MMP‐9* rs2250889.

Genomic DNA from peripheral blood was extracted using a modified phenol/chloroform method and purified using the UNIQ‐10 kit (Sangon Biotech Co., Ltd.). The genotyping of these SNPs was performed by authors blinded to the clinical data of patients, using the matrix‐assisted laser desorption/ionization time of flight (MALDI‐TOF) mass spectrometry method, as our previously described (Yi et al., 2015). In brief, each SNP gene possessed a specific genotype, with two amplification primers, and one extension primer. The reaction mix was desalted by adding 6 mg of cation exchange resin (Sequenom Inc.), mixed, and resuspended in 25 µl of water. Once the primer extension reaction was completed, the samples were spotted onto a 384‐well spectroCHIP (Sequenom Inc.) using MassARRAY Nanodispenser (Sequenom Inc.) and genotyped using the MALDI‐TOF mass spectrometer. Genotype calling was performed in real time with MassARRAY RT software version 3.0.0.4 and analyzed using the MassARRAY Typer software version 3.4 (Sequenom Inc.).

### 2.6 Statistical analysis

The data were analyzed using SPSS 16.0 software. The results are expressed as percentages for categorical variables, and continuous variables are expressed as mean ± *SD*. Baseline clinical characteristics and genotype distribution of the nine variants were compared using Student's *t* test (continuous variables) and chi‐square test (categorical variables) between patients with and without ND.

The allele frequencies for Hardy–Weinberg equilibrium were evaluated using chi‐square test. The GMDR software was used to assess gene–gene interactions under various scenarios as previously reported (Lou et al., 2007; Yi et al., 2015). In brief, the nine variants were coded from number 1 to 9, and GMDR computes the maximum‐likelihood estimates and the scores of all individuals under the null hypothesis. The cumulative score is calculated within each multifactor cell, which is labeled either as high risk if the average score meets or exceeds a pre‐assigned threshold of 0 or as low‐risk if the score is less than 0. An exhaustive search of all possible one‐ to ten‐locus models was performed for all variants. The model with the minimum prediction error, the maximum cross‐validation consistency score, and 0.05 or lower *p* value derived from the sign test automatically in the GMDR software was considered as the best model, which were confirmed by a permutation test implemented in the GMDR software as well.

Incidence of ND between patients with and without high‐risk interactive genotype was compared by chi‐square test. The high‐risk interaction genotype was assigned as one, and low‐risk interaction genotype was assigned as zero in Cox proportional‐hazards model and multivariable logistic regression analysis. The independent contribution of gene–gene interaction to ND was assessed using Cox proportional‐hazards model after adjusting for covariates (variables with *p* value < .2 by univariate analysis) and reported as the hazard ratio (HR) with the 95% confidence interval (CI). The influence of the high‐risk interactive genotype on functional outcome was investigated by multivariable logistic regression analysis, after adjusting for the main baseline variables related to each main variable in the univariate analysis (enter approach and probability of entry *p* < .05) and reported as odds ratio (OR) with 95% CI.

All tests were two‐sided, and *p* value < .05 was considered statistically significant.

## 3 RESULTS

### 3.1 Clinical characteristics in patients with and without ND

**Figure 1:**
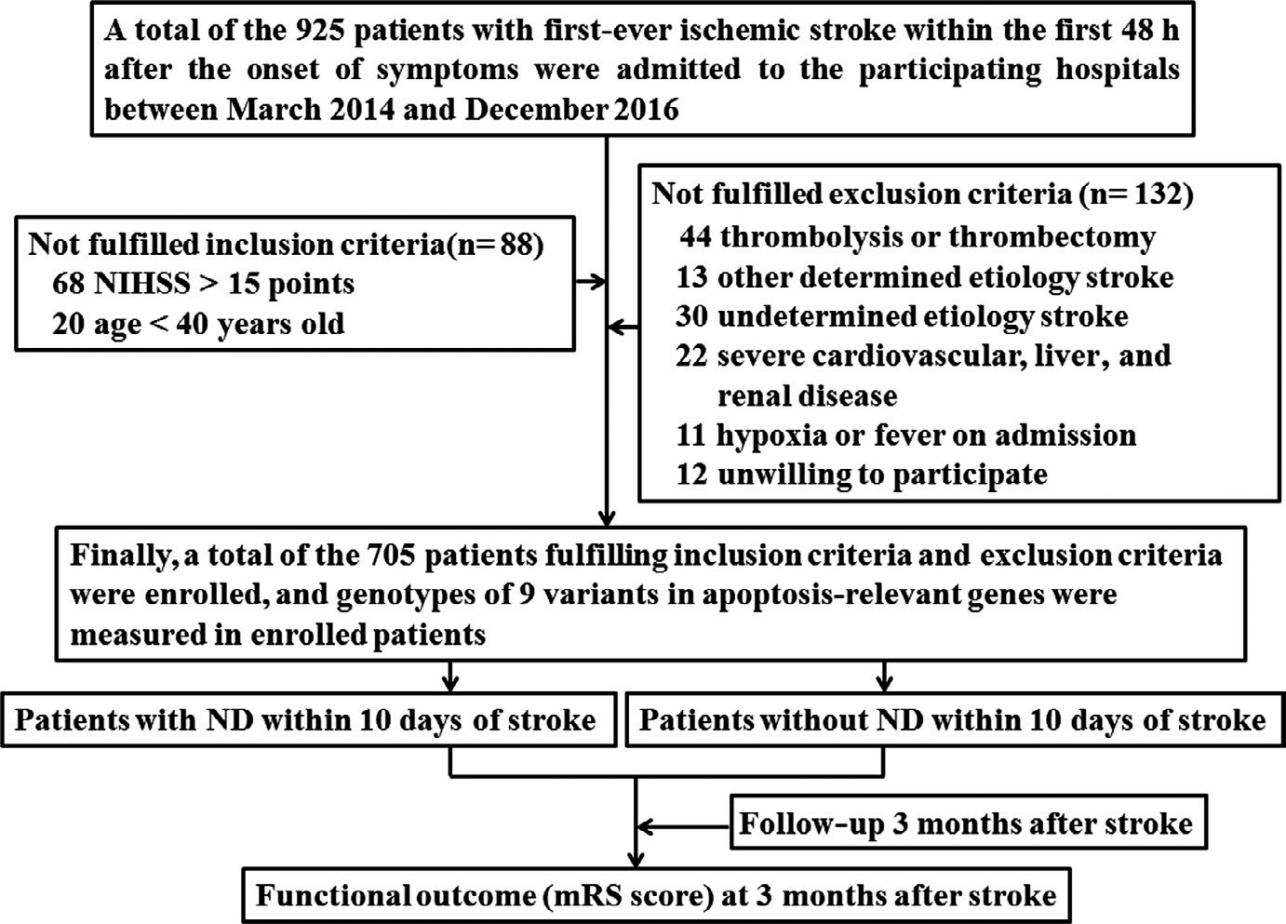
Flowchart in this study

Between March 2014 and December 2016, 925 patients with first‐ever IS within the first 48 hr after the onset of symptoms were admitted to the participating hospitals. Among the 925 patients, 220 patients did not fulfill inclusion criteria and exclusion criteria. Finally, a total of the 705 patients fulfilling inclusion criteria and exclusion criteria were enrolled. The detailed procedure in this study was presented in Figure 1. Among the 705 enrolled patients, the duration of in‐hospital ranged from 10 to 18 days (median, 13.6 days). There were no patients discharged within 10 days after stroke onset. ND occurred in 174 (24.7%) patients within the first 10 days of stroke onset. Compared with patients without ND, the age was older, and fasting glucose and hemoglobin A1c were higher in patients with ND (Table 1).

**Table 1:** Baseline characteristics in patients with and without ND

Characteristics	Patients with ND (*n* = 174)	Patients without ND (*n* = 531)	*p* Value
Age (years)	70.8 ± 14.3	68.2 ± 14.7	.043
Men (*n*, %)	100 (57.5)	295 (55.6)	.673
Hypertension (*n*, %)	140 (80.5)	411 (77.4)	.412
Diabetes mellitus (*n*, %)	65 (37.4)	161 (30.3)	.091
Atrial fibrillation (*n*, %)	21 (12.1)	58 (10.9)	.671
Current smoker (*n*, %)	72 (41.4)	213 (40.1)	.778
Hyperlipidemia (*n*, %)	100 (57.5)	289 (54.4)	.482
Systolic blood pressure (mmHg)	153.4 ± 14.8	152.6 ± 17.5	.552
Diastolic blood pressure (mm Hg)	90.1 ± 11.8	89.5 ± 15.7	.593
Fasting glucose (mM)	7.5 ± 2.7	6.6 ± 2.5	<.001
Hemoglobin A1c (%)	7.3 ± 2.4	6.5 ± 2.2	<.001
Onset to admission time (h)	29.9 ± 16.2	30.4 ± 17.8	.731
NIHSS score at admission	10.9 ± 3.5	10.4 ± 3.7	.125
Stroke subtype (*n*, %)			
Atherothrombosis	102 (58.6)	306 (57.6)	.986
Small artery disease	41 (23.6)	134 (25.2)	
Cardioembolism	31 (17.8)	91 (17.1)	
In‐hospital treatment (*n*, %)			
Antihypertensive drugs	145 (83.3)	422 (79.5)	.296
Hypoglycemic drugs	69 (39.7)	171 (32.2)	.089
Statins	166 (95.4)	519 (97.7)	.113
Aspirin	107 (61.5)	337 (63.5)	.526
Aspirin plus clopidogrel	44 (25.3)	168 (31.6)	.111

Abbreviations: ND, neurologic deterioration; NIHSS, National Institutes of Health Stroke Scale.

### 3.2 Genotype distributions in patients with and without ND

The genotype distributions of the nine variants were consistent with the Hardy–Weinberg equilibrium (all *p* > .05). There were no significant differences of genotype distributions in the nine variants between patients with and without ND by univariate analysis (*p* > .05 for each variant individually, Table 2).

**Table 2:** Genotype distribution comparison between patients with and without ND (%)

	Patients with ND (*n* = 174)	Patients without ND (*n* = 531)	*p*‐Value
*P53* (rs1042522)			
CC	27 (15.5)	97 (18.3)	.738
CG	88 (50.6)	263 (49.5)	
GG	59 (33.9)	171 (32.2)	
*MDM‐2* (rs2279744)			
TT	36 (20.7)	101 (19.0)	.885
TG	90 (51.7)	275 (51.8)	
GG	48 (27.6)	155 (29.2)	
*MDM‐2* (rs1690916)			
GG	93 (53.4)	307 (57.8)	.294
GA	65 (37.4)	194 (36.5)	
AA	16 (9.2)	30 (5.6)	
*P21* (rs1801270)			
CC	38 (21.8)	134 (25.2)	.366
CA	82 (47.1)	257 (48.4)	
AA	54 (31.0)	140 (26.4)	
*MMP‐9* (rs1056628)			
AA	114 (65.5)	364 (68.5)	.516
AC	48 (27.6)	142 (26.7)	
CC	12 (6.9)	25 (4.7)	
*MMP‐9* (rs3918242)			
CC	116 (66.7)	388 (73.1)	.188
CT	46 (26.4)	121 (22.8)	
TT	12 (6.9)	22 (4.1)	
*MMP‐9* (rs17576)			
AA	20 (11.5)	60 (11.3)	.893
AG	70 (40.2)	203 (38.2)	
GG	84 (48.3)	268 (50.5)	
*MMP‐9* (rs3787268)			
AA	65 (37.4)	218 (41.1)	.678
AG	50 (28.7)	145 (27.3)	
GG	59 (33.9)	168 (31.6)	
*MMP‐9* (rs2250889)			
CC	99 (56.9)	296 (55.7)	.725
CG	52 (29.9)	173 (32.6)	
GG	23 (13.2)	62 (11.7)	

Abbreviation: ND, neurologic deterioration.

### 3.3 Gene–gene interactions

Although the nine variants in apoptotic‐relevant genes were not significantly associated with ND by univariate analysis, there was a gene–gene interaction among the nine variants using GMDR analysis. The best model for ND was interaction among *P53* rs1042522, *MDM‐2* rs2279744, and *MMP‐9*rs3918242 after adjusting for confounding variables (*p* = .021, Table 3). The one‐locus model was computed for each variant and the empirical *p* values for prediction error using permutation testing were .026, indicating the interactions among the three variants synergistically contributed to a higher risk of ND than did single variant alone.

**Table 3:** Gene–gene interaction identified by GMDR analysis for ND

Best model^6^	Training balanced accuracy	Testing balanced accuracy	Cross‐validation consistency	Sign test (*p* value)
1	0.523	0.486	7/10	8 (.421)
1,2	0.581	0.523	8/10	7 (.227)
1,2,3	0.632	0.597	10/10	9 (.021)
1,2,4,5	0.504	0.468	9/10	6 (.137)
1,2,3,4,5	0.584	0.467	6/10	8 (.359)
1,2,3,4,5,6	0.611	0.543	5/10	6 (.554)
1,2,3,4,5,6,7	0.425	0.611	7/10	6 (.472)
1,2,3,4,5,6,7,8	0.542	0.493	9/10	6 (.535)
1,2,3,4,5,6,7,8,9	0.798	0.522	8/10	6 (.633)

Abbreviations: GMDR, generalized multifactor dimensionality reduction; ND, neurologic deterioration.

Numbers 1–9 represent rs1042522, rs2279744, rs3918242, rs1690916, rs1801270, rs1056628, rs3787268, rs17576, and rs2250889, respectively.

### 3.4 Associations between different genotype combinations and ND risk

Then, we evaluated the relationship of different genotype combinations of the three interactive variants with the risk of ND. The wild‐type genotype for the three variants was used as the reference. Compared to the patients harboring wild‐type genotype rs1042522CC, rs2279744TT, and rs3918242CC, the relative risk of different genotype combinations among the three variants for ND was assessed. The risks for ND were higher in patients harboring rs1042522GG, rs2279744GG, and rs3918242TT; rs1042522GG, rs2279744GG, and rs3918242TT/CT; and rs1042522GG, rs2279744TG, and rs3918242CT, compared with those carrying rs1042522CC, rs2279744TT, and rs3918242CC (Table 4). The three combination genotypes of rs1042522, rs2279744, and rs3918242 were defined as high‐risk interactive genotype. The other combination genotypes of rs1042522, rs2279744, and rs3918242 did not reach statistical significance level of 0.05 (Table 4) and were considered as low‐risk interactive genotype.

**Table 4:** Associations between genotype combinations and ND

Rs1042522	CC	GG	GG	GG	CG	GG, CG	CG	GG, CG
Rs2279744	TT	GG	GG	TG	TG	GG	GG, TG	GG, TG
Rs3918242	CC	TT	TT, CT	CT	CT	TT	TT	TT, CT
OR	1^8^	2.76	2.12	1.89	1.52	1.24	1.13	1.08
95% CI	—	1.31–5.96	1.18–5.16	1.06–4.58	0.97–2.36	0.95–2.88	0.86–2.05	0.83–1.95
*p* Value	—	.003	.008	.027	.198	.387	.497	.586

Abbreviations: CI, confidence interval; ND, neurologic deterioration; OR, odds ratio.

The wild‐type genotype for each variant was used as the reference.

### 3.5 Association of high‐risk interactive genotype with risk of ND

The incidence of ND was significantly higher in patients carrying high‐risk interactive genotype than those carrying low‐risk interactive genotype (33.2% [65/196] vs. 21.4% [109/509], *p* < .001). The risk for ND conferred by high‐risk interactive genotype was evaluated using Cox proportional‐hazards model. The high‐risk interaction was assigned as one, and low‐risk interaction was assigned as zero. The other predictors with *p* value < .2 by univariate analysis also entered in Cox proportional‐hazards model for ND, including age, diabetes mellitus, fasting blood glucose, hemoglobin A1c, NIHSS score at admission, statins, aspirin plus clopidogrel, and rs3918242CT/TT. The results showed the high‐risk interaction among rs1042522, rs2279744, and rs3918242 was independently associated with higher risk of ND after adjusting for the covariates (HR, 2.04, 95% CI: 1.22–5.64, *p* = .018, Table 5).

**Table 5:** Cox regression analysis of independent predictors for ND

Factor	HR	95% CI	*p* Value
Age	0.85	0.72–1.46	.453
Diabetes mellitus	0.91	0.85–1.95	.322
Fasting blood glucose	1.73	1.02–2.76	.041
Hemoglobin A1c	1.34	0.96–1.86	.106
NIHSS score at admission	1.21	0.92–2.13	.412
Statins	0.86	0.68–1.22	.633
Aspirin plus clopidogrel	0.72	0.53–0.99	.033
Rs3918242CT/TT	1.36	0.92–2.36	.286
High‐risk interactive variable	2.04	1.22–5.64	.018

Note

HR for continuous variables means per 1 − *SD* increase.

Abbreviations: CI, confidence interval; HR, hazard ratio; ND, neurologic deterioration.

### 3.6 Association of high‐risk interactive genotype with poor functional outcome

Using the mRS to evaluate the disability or dependence in daily living activities of stroke victims at 3 months, we found 184 (26.1%) patients were poor functional outcome (mRS score > 2). There were no significant differences in poor functional outcome among genotypes of the 9 variants. However, the percentage of poor functional outcome was significantly higher in patients carrying the high‐risk interactive genotype than those carrying the low‐risk interactive genotype (35.6% [84/236] vs. 21.3% [100/469], *p* < .001). After adjustment for the covariates, including age, diabetes mellitus, hypertension, fasting blood glucose, NIHSS score at admission, neurologic deterioration, and aspirin plus clopidogrel, the high‐risk interaction among rs1042522, rs2279744, and rs3918242 was an independent predicting marker of poor functional outcome (OR, 2.68, 95% CI: 1.68–7.86, *p* = .004, Table 6), as revealed by multivariate logistic regression analysis.

**Table 6:** Logistic regression analysis independent variables associated with poor functional outcome at 3 months (mRS > 2) after admission

Factor	OR	95% CI	*p* Value
Age	0.87	0.68–1.42	.637
Diabetes mellitus	0.86	0.72–1.17	.623
Hypertension	1.01	0.92–1.85	.564
NIHSS scores on admission	1.82	1.06–3.46	.024
Neurologic deterioration	3.68	1.94–9.68	.002
Fasting blood glucose	1.04	0.94–2.35	.352
Aspirin plus clopidogrel	0.86	0.67–1.02	.132
High‐risk interactive variable	2.68	1.68–7.86	.004

Note

OR for continuous variables means per 1 − *SD* increase.

Abbreviations: CI, confidence interval; mRS, modified Rankin Scale; NIHSS, National Institutes of Health Stroke Scale; OR, odds ratio.

## 4 DISCUSSION

In this study, we found that 174 (24.7%) patients suffered from ND, and 184 (26.1%) patients were poor functional outcome (mRS score > 2). Although the nine variants in apoptotic‐relevant genes were not associated with ND and functional outcome by univariate analysis, GMDR analysis revealed that there was a gene–gene interaction among *P53* rs1042522, *MDM‐2* rs2279744, and *MMP‐9* rs3918242, and the high‐risk interaction among the three variants was independently associated with higher risk of ND and poor functional outcome.

Some studies have revealed that low circulating levels of retinoic acid and plasma neuroendocrine biomarkers, including baseline plasma brain natriuretic peptide, N‐terminal pro‐brain natriuretic peptide, and cortisol and copeptin levels on admission can predict outcomes and mortality after acute IS (Tu, Dong, Zhao, Yang, & Chen, 2013; Tu et al., 2019). Neuronal apoptosis is an important mechanism of delayed ischemic brain damage in animal experiments (Sairanen et al., 2006). The presence of apoptotic neurons in the ischemic penumbra is associated with poor functional prognosis and mortality after acute IS (Gomez‐Sanchez et al., 2011). p53‐mediated neuronal death plays a central role of stroke pathophysiology in a mouse model of focal permanent cerebral ischemia (Liu et al., 2013). However, the possible role of SNPs of p53 apoptotic pathway relevant genes in ND and functional outcome after IS has not been thoroughly understood.

The detailed information of the 9 SNPs in this study was summarized in Table 7. Extensive evidences have shown that SNPs of *P53* and *MDM2* rs2279744 play important roles in DNA damage and cell apoptosis and were independently related to high risk of cancer (Chen et al., 2015; Duan et al., 2018; Liu et al., 2011). Human *P*53 Arg72Pro (rs1042522) SNP controls susceptibility to ischemia‐induced neuronal apoptosis and the functional outcome after stroke (Gomez‐Sanchez et al., 2011). *P*53 rs1042522 SNP can condition neuronal ischemic tolerance by modulating mitochondrial p53 stabilization (Ramos‐Araque et al., 2018). *MMP‐9* polymorphisms regulate the transcription of MMP‐9 protein and are associated with increased IS or cancer risk and hemorrhagic transformation of IS (Yuan et al., 2013; Zhang, Cao, Xu, Li, & Xu, 2015; Zhu et al., 2018). In this study, we did not find the association of nine variants in apoptotic‐relevant genes with ND and functional outcome by univariate analysis. However, the most noteworthy finding in this study was that there was a gene–gene interaction among rs1042522, rs2279744, and rs3918242 using GMDR analysis, and the high‐risk interaction among the three variants was independently associated with risk of ND and poor functional outcome. This indicates interaction among the three variants synergistically contributes to a higher risk of ND and poor functional outcome than do single variant alone.

**Table 7:** Detailed information of the 9 SNPs

	Chromosomal location	Types of amino acid changes	Functional consequence	Results of studies or clinical significance:
*P53*(rs1042522)	17:7676154	Pro72Arg	Missense variant	Uncertain‐significance or drug‐response
*MDM‐2*(rs2279744)	12:68808800	N/A	Intron variant	Accelerated tumor formation and risk
*MDM‐2*(rs1690916)	12:68841626	N/A	3 Prime UTR variant	Not Reported in Clin
*P21*(rs1801270)	6:36684194	Ser31Arg	Missense variant	Tumor risk
*MMP‐9*(rs1056628)	20:46016407	N/A	Intron variant and 3 Prime UTR variant	Not Reported in Clin
*MMP‐9*(rs3918242)	20:46007337	N/A	2KB upstream variant	Not reported in Clin
*MMP‐9*(rs17576)	20:46011586	Gln279Arg	Missense variant	Metaphyseal anadysplasia
*MMP‐9*(rs3787268)	20:46013092	N/A	Intron variant	Not Reported in Clin
*MMP‐9*(rs2250889)	20:46013767	Arg574Leu	Missense variant	Metaphyseal anadysplasia

Abbreviation: SNPs, single nucleotide polymorphisms.

The nature of the gene–gene interaction among the three variants is not clear. It may be due to rs1042522, rs2279744 and rs3918242 may synergistically affect cell‐cycle regulation, DNA damage, cell apoptosis, and BBB destruction (Chen et al., 2015; Duan et al., 2018; Gomez‐Sanchez et al., 2011; Yousefi et al., 2014), which contribute to a higher risk of ND and poor functional outcome after IS. In previous reports, *P53* encodes p53 tumor suppressor protein that can mediate apoptosis in eukaryotic cells (Yousefi et al., 2014), and it is a tumor suppressor gene involved in the G1‐S checkpoint and has the function of gene guarding (Pietsch, Humbey, & Murphy, 2006). *P*53 rs1042522 SNP occurs in a proline‐rich domain involved in the proapoptotic function of p53 (Pietsch et al., 2006; Sakamuro, Sabbatini, White, & Prendergast, 1997). Variant of *P*53 rs1042522 is a potent inducer of apoptosis and inhibitor of oncogenic transformation and determines the age of onset and cancer progression (Pietsch et al., 2006; Whibley, Pharoah, & Hollstein, 2009; Zhu et al., 2010). Gomez‐Sanchez et al. (2011) reported *P*53 *Arg/Arg* genotype was linked to early ND and poor functional outcome after IS. In primary cultured neurons, Arg72‐p53 interacted directly with mitochondrial B‐cell lymphoma‐extra large activated the intrinsic apoptotic pathway and ischemia‐induced apoptotic cell death. Delayed treatment with a p53 inhibitor (pifithrin‐alpha) may modify stroke‐induced endogenous neurogenesis and improve the functional recovery in stroke animals (Luo et al., 2009). *MDM‐2* is an important regulator of *P53* and has the function of degrading *P53* (Qiu et al., 2008). Although there was no report regarding the relationship between *MDM‐2* genetic polymorphisms and ND or functional outcome after IS, some studies have shown that *MDM‐2* polymorphisms may be a risk factor for uterine fibroids and hepatocellular carcinoma (Dong et al., 2012; Salimi et al., 2015). This was in good agreement with the highlighted notion that neuronal death and oncogenesis may share common mechanistic foundations (Morris, Veeriah, & Chan, 2010). MMP‐9 can activate numerous pro‐inflammatory cytokines and chemokines and involves in neuronal damage and apoptosis (Barr et al., 2010; Candelario‐Jalil et al., 2009). Previous studies showed that *MMP‐9* polymorphisms were associated with increased risk of IS, cancer, and hemorrhagic transformation of IS (Yuan et al., 2013; Zhang et al., 2015; Zhu et al., 2018). Up to date, there were no studies to investigate the association of interaction among the SNPs of *P53*, *P21*, *MDM‐2*, and *MMP‐9* with ND or functional outcome after IS. However, Duan et al. (2018) has revealed that interaction among *P53*, *P21*, and *MDM‐2* may affect cholinesterase activity in people with exposure to omethoate. Thus, we reason that the high‐risk interaction among *P53*, *MDM‐2*, and *MMP‐9* could synergistically activate the intrinsic apoptotic pathway and ischemia‐induced apoptotic cell death, thereby increasing the risk of ND and poor functional outcome after IS.

Despite our findings are interesting, there are several limitations in this study. First, this study was performed in two‐centers, and the samples were small. Thus, our findings should be validated in larger samples, multicenter studies. Second, although we genotyped known functional variants in *P53*, *P21*, *MDM‐2*, and *MMP‐9* gene, some rare functional variants were not assessed in this population. Furthermore, apoptotic pathways are very complex, and many genes may involve in regulating apoptosis. In this study, we only investigated the association of nine variants in P53 apoptotic pathway with ND and functional outcome. Thus, future studies involving a larger set of genetic variants must be conducted to investigate the full extent of gene–gene interaction effect on ND and functional outcome. Third, although we found the three variants in *P53*, *MDM‐2*, and *MMP‐9* could synergistically contribute to a higher risk of ND and poor functional outcome, we did not investigate the molecular mechanisms of the gene–gene interactions. Therefore, in the next study we will plan to use the primary cultured neurons or animal models of cerebral ischemia to explain the molecular mechanisms of interaction among the three variants. Finally, lack of an independent sample for replication was also a limitation in this study.

## 5 CONCLUSION

The incidence of ND and poor functional outcome after stroke is very common. There is a gene–gene interaction among *P53* rs1042522, *MDM‐2* rs2279744, and *MMP‐9* rs3918242. The high‐risk interaction among the three variants may increase the risk of ND and poor functional outcome and may be considered as a genetic marker of predicting ND and poor functional outcome after IS.

## CONFLICT OF INTEREST

The authors declare that there are no conflicts of interest.

## Data Availability

The data that support the findings of this study are available on request from the corresponding author. The data are not publicly available due to privacy or ethical restrictions.

## References

[brb31492-bib-0001] Banks, J. L. , & Marotta, C. A. (2007). Outcomes validity and reliability of the modified Rankin scale: Implications for stroke clinical trials: A literature review and synthesis. Stroke, 38, 1091–1096. 10.1161/01.STR.0000258355.23810.c6 17272767

[brb31492-bib-0002] Barr, T. L. , Latour, L. L. , Lee, K. Y. , Schaewe, T. J. , Luby, M. , Chang, G. S. , … Warach, S. (2010). Blood‐brain barrier disruption in humans is independently associated with increased matrix metalloproteinase‐9. Stroke, 41, e123–e128. 10.1161/STROKEAHA.109.570515 20035078 PMC2827673

[brb31492-bib-0003] Broughton, B. R. , Reutens, D. C. , & Sobey, C. G. (2009). Apoptotic mechanisms after cerebral ischemia. Stroke, 40, e331–e339. 10.1161/STROKEAHA.108.531632 19182083

[brb31492-bib-0004] Candelario‐Jalil, E. , Yang, Y. , & Rosenberg, G. A. (2009). Diverse roles of matrix metalloproteinases and tissue inhibitors of metalloproteinases in neuroinflammation and cerebral. Neuroscience, 158, 983–994. 18621108 10.1016/j.neuroscience.2008.06.025 PMC3584171

[brb31492-bib-0005] Castillo, J. (1999). Deteriorating stroke: Diagnostic criteria, predictors, mechanisms and treatment. Cerebrovascular Disease, 9, 1–8. 10.1159/000047548 10436319

[brb31492-bib-0006] Chen, R. , Liu, S. , Ye, H. , Li, J. , Du, Y. , Chen, L. , … Yang, H. (2015). Association of p53 rs1042522, MDM2 rs2279744, and p21 rs1801270 polymorphisms with retinoblastoma risk and invasion in a Chinese population. Scientific Reports, 5, 13300. 26289323 10.1038/srep13300 PMC4642541

[brb31492-bib-0007] Dong, D. , Gao, X. , Zhu, Z. , Yu, Q. , Bian, S. , & Gao, Y. (2012). 2012 A 40‐bp insertion/deletion polymorphism in the constitutive promoter of MDM2 confers risk for hepatocellular carcinoma in a Chinese population. Gene, 497, 66–70. 10.1016/j.gene.2012.01.004 22285926

[brb31492-bib-0008] Duan, X. , Yang, Y. , Wang, S. , Feng, X. , Wang, T. , Wang, P. , … Wang, W. (2017). Changes in the expression of genes involved in cell cycle regulation and the relative telomere length in the process of canceration induced by omethoate. Tumour Biology, 39, 1010428317719782. 10.1177/1010428317719782 28718371

[brb31492-bib-0009] Duan, X. , Yang, Y. , Wang, S. , Feng, X. , Wang, T. , Wang, P. , … Wang, W. (2018). Interaction between polymorphisms in cell‐cycle genes and environmental factors in regulating cholinesterase activity in people with exposure to omethoate. Royal Society Open Science, 5, 172357. 10.1098/rsos.172357 29892419 PMC5990798

[brb31492-bib-0010] Gomez‐Sanchez, J. C. , Delgado‐Esteban, M. , Rodriguez‐Hernandez, I. , Sobrino, T. , Perez de la Ossa, N. , Reverte, S. , … Almeida, A. (2011). The human Tp53 Arg72Pro polymorphism explains different functional prognosis in stroke. Journal of Experimental Medicine, 208, 429–437. 10.1084/jem.20101523 PMC3058581 21357744

[brb31492-bib-0011] Guan, T. , Ma, J. , Li, M. , Xue, T. , Lan, Z. , Guo, J. , … Liu, Y. (2017). Rapid transitions in the epidemiology of stroke and its risk factors in China from 2002 to 2013. Neurology, 89, 53–61. 10.1212/WNL.0000000000004056 28566547

[brb31492-bib-0012] Han, S. W. , Kim, S. H. , Lee, J. Y. , Chu, C. K. , Yang, J. H. , Shin, H. Y. , … Heo, J. H. (2007). A new subtype classification of ischemic stroke based on treatment and etiologic mechanism. European Neurology, 57, 96–102. 10.1159/000098059 17179712

[brb31492-bib-0013] Jauch, E. C. , Saver, J. L. , Adams, H. P. Jr , Bruno, A. , Connors, J. J. , Demaerschalk, B. M. , … Tirschwell, D. L. (2013). Guidelines for the early management of patients with acute ischemic stroke: A guideline for healthcare professionals from the American Heart Association/American Stroke Association. Stroke, 44, 870–947. 23370205 10.1161/STR.0b013e318284056a

[brb31492-bib-0014] Karimian, A. , Ahmadi, Y. , & Yousefi, B. (2016). Multiple functions of p21 in cell cycle, apoptosis and transcriptional regulation after DNA damage. DNA Repair, 42, 63–71. 10.1016/j.dnarep.2016.04.008 27156098

[brb31492-bib-0015] Kernan, W. N. , Ovbiagele, B. , Black, H. R. , Bravata, D. M. , Chimowitz, M. I. , Ezekowitz, M. D. , … Wilson, J. A. (2014). Guidelines for the prevention of stroke in patients with stroke and transient ischemic attack: A guideline for healthcare professionals from the American Heart Association/American Stroke Association. Stroke, 45, 2160–2236. 10.1161/STR.0000000000000024 24788967

[brb31492-bib-0016] Liu, F. , Li, B. , Wei, Y. , Chen, X. , Ma, Y. , Yan, L. , & Wen, T. (2011). P21 codon 31 polymorphism associated with cancer among white people: Evidence from a meta‐analysis involving 78,074 subjects. Mutagenesis, 26, 513–521. 10.1093/mutage/ger010 21415438

[brb31492-bib-0017] Liu, X. , Li, F. , Zhao, S. , Luo, Y. , Kang, J. , Zhao, H. , … Ji, X. (2013). MicroRNA‐124–mediated regulation of inhibitory member of apoptosis‐stimulating protein of p53 family in experimental stroke. Stroke, 44, 1973–1980. 10.1161/STROKEAHA.111.000613 23696548

[brb31492-bib-0018] Lou, X.‐Y. , Chen, G.‐B. , Yan, L. , Ma, J. Z. , Zhu, J. , Elston, R. C. , & Li, M. D. (2007). A generalized combinatorial approach for detecting gene‐by‐gene and gene‐by‐environment interactions with application to nicotine dependence. American Journal of Human Genetics, 80, 1125–1137. 10.1086/518312 17503330 PMC1867100

[brb31492-bib-0019] Luo, Y. , Kuo, C. C. , Shen, H. , Chou, J. , Greig, N. H. , Hoffer, B. J. , & Wang, Y. (2009). Delayed treatment with a p53 inhibitor enhances recovery in stroke brain. Annals of Neurology, 65, 520–530. 10.1002/ana.21592 19475672 PMC2690614

[brb31492-bib-0020] Macleod, K. F. , Sherry, N. , Hannon, G. , Beach, D. , Tokino, T. , Kinzler, K. , … Jacks, T. (1995). p53‐dependent and independent expression of p21 during cell growth, differentiation, and DNA damage. Genes & Development, 9, 935–944. 10.1101/gad.9.8.935 7774811

[brb31492-bib-0021] Morris, L. G. , Veeriah, S. , & Chan, T. A. (2010). Genetic determinants at the interface of cancer and neurodegenerative disease. Oncogene, 29, 3453–3464. 10.1038/onc.2010.127 20418918 PMC3005561

[brb31492-bib-0022] Moumen, A. , Patane, S. , Porras, A. , Dono, R. , & Maina, F. (2007). Met acts on Mdm2 via mTOR to signal cell survival during development. Development, 134, 1443–1451. 10.1242/dev.02820 17329361

[brb31492-bib-0023] Pietsch, E. C. , Humbey, O. , & Murphy, M. E. (2006). Polymorphisms in the p53 pathway. Oncogene, 25, 1602–1611. 10.1038/sj.onc.1209367 16550160

[brb31492-bib-0024] Qiu, Y. L. , WangW, W. T. , Liu, J. , Sun, P. , Qian, J. , Jin, L. , & Xia, Z.‐L. (2008). Genetic polymorphisms, messenger RNA expression of p53, p21, and CCND1, and possible links with chromosomal aberrations in Chinese vinyl chloride‐exposed workers. Cancer Epidemiology, Biomarkers & Prevention, 17, 2578–2584. 10.1158/1055-9965.EPI-07-2925 18842998

[brb31492-bib-0025] Ramos‐Araque, M. E. , Rodriguez, C. , Vecino, R. , Cortijo Garcia, E. , de Lera‐Alfonso, M. , Sanchez Barba, M. , … Delgado‐Esteban, M. (2018). The neuronal ischemic tolerance is conditioned by the Tp53 Arg72Pro polymorphism. Translational Stroke Research, 10, 204–215. 10.1007/s12975-018-0631-1 29687302 PMC6421278

[brb31492-bib-0026] Sairanen, T. , Karjalainen‐Lindsberg, M. L. , Paetau, A. , Ijäs, P. , & Lindsberg, P. J. (2006). Apoptosis dominant in the periinfarct area of human ischaemic stroke – A possible target of antiapoptotic treatments. Brain, 129, 189–199. 10.1093/brain/awh645 16272167

[brb31492-bib-0027] Sakamuro, D. , Sabbatini, P. , White, E. , & Prendergast, G. C. (1997). The polyproline region of p53 is required to activate apoptosis but not growth arrest. Oncogene, 15, 887–898. 9285684 10.1038/sj.onc.1201263

[brb31492-bib-0028] Salimi, S. , Hajizadeh, A. , Khodamian, M. , Pejman, A. , Fazeli, K. , & Yaghmaei, M. (2015). Age‐dependent association of MDM2 promoter polymorphisms and uterine leiomyoma in South‐East Iran: A preliminary report. Journal of Obstetrics and Gynaecology Research, 41, 729–734. 10.1111/jog.12625 25511444

[brb31492-bib-0029] Tu, W. J. , Dong, X. , Zhao, S. J. , Yang, D. G. , & Chen, H. (2013). Prognostic value of plasma neuroendocrine biomarkers in patients with acute ischaemic stroke. Journal of Neuroendocrinology, 25, 771–778. 10.1111/jne.12052 23701638

[brb31492-bib-0030] Tu, W. J. , Qiu, H. C. , Zhang, Y. , Cao, J. L. , Wang, H. , Zhao, J. Z. , … Zeng, X. (2019). Lower serum retinoic acid level for prediction of higher risk of mortality in ischemic stroke. Neurology, 92, e1678–e1687. 10.1212/WNL.0000000000007261 30850446

[brb31492-bib-0031] Vahidy, F. S. , Hicks, W. J. 2nd , Acosta, I. , Hallevi, H. , Peng, H. , Pandurengan, R. , … Savitz, S. I. (2014). Neurofluctuation in patients with subcortical ischemic stroke. Neurology, 83, 398–405. 10.1212/WNL.0000000000000643 24966405 PMC4132571

[brb31492-bib-0032] van Swieten, J. C. , Koudstaal, P. J. , Visser, M. C. , Schouten, H. J. , & van Gijn, J. (1988). Interobserver agreement for the assessment of handicap in stroke patients. Stroke, 19, 604–607. 10.1161/01.STR.19.5.604 3363593

[brb31492-bib-0033] Weimar, C. , Ziegler, A. , König, I. R. , & Diener, H. C. (2002). Predicting functional outcome and survival after acute ischemic stroke. Journal of Neurology, 249, 888–895. 10.1007/s00415-002-0755-8 12140674

[brb31492-bib-0034] Whibley, C. , Pharoah, P. D. , & Hollstein, M. (2009). p53 polymorphisms: Cancer implications. Nature Reviews Cancer, 9, 95–107. 19165225 10.1038/nrc2584

[brb31492-bib-0035] Yagnik, G. , Jahangiri, A. , Chen, R. , Wagner, J. R. , & Aghi, M. K. (2017). Role of a p53 polymorphism in the development of nonfunctional pituitary adenomas. Molecular and Cellular Endocrinology, 446, 81–90. 10.1016/j.mce.2017.02.017 28214592 PMC5553295

[brb31492-bib-0036] Yi, X. , Han, Z. , Zhou, Q. , Lin, J. , & Liu, P. (2016). 20‐hydroxyeicosatetraenoic acid as a predictor of neurological deterioration in acute minor ischemic stroke. Stroke, 47, 3045–3047. 10.1161/STROKEAHA.116.015146 27834744

[brb31492-bib-0037] Yi, X. , Liao, D. , Fu, X. , Zhang, B. , & Wang, C. (2015). Interaction among CYP2C8, EPHX2, and CYP4A11 gene variants significantly increases the risk for ischemic stroke in Chinese populations. Journal of Atherosclerosis and Thrombosis, 22, 1148–1157. 25947240 10.5551/jat.29025

[brb31492-bib-0038] Yousefi, B. , Rahmati, M. , & Ahmadi, Y. (2014). The roles of p53R2 in cancer progression based on the new function of mutant p53 and cytoplasmic p21. Life Sciences, 99, 14–17. 10.1016/j.lfs.2014.01.063 24486301

[brb31492-bib-0039] Yuan, M. , Zhan, Q. , Duan, X. , Song, B. , Zeng, S. , Chen, X. , … Xia, J. (2013). A functional polymorphism at miR‐491‐5p binding site in the 3'‐UTR of MMP‐9 gene confers increased risk for atherosclerotic cerebral infarction in a Chinese population. Atherosclerosis, 226, 447–452. 10.1016/j.atherosclerosis.2012.11.026 23257658

[brb31492-bib-0040] Zhang, X. , Cao, X. , Xu, X. , Li, A. , & Xu, Y. (2015). Correlation between the ‐1562C/T polymorphism in the matrix metalloproteinase‐9 gene and hemorrhagic transformation of ischemic stroke. Experimental and Therapeutic Medicine, 9, 1043–1047. 10.3892/etm.2015.2186 25667675 PMC4316928

[brb31492-bib-0041] Zhu, F. , Dollé, M. E. , Berton, T. R. , Kuiper, R. V. , Capps, C. , Espejo, A. , … Johnson, D. G. (2010). Mouse models for the p53 R72P polymorphism mimic human phenotypes. Cancer Research, 70, 5851–5859. 10.1158/0008-5472.CAN-09-4646 20587514 PMC2905499

[brb31492-bib-0042] Zhu, P. , Liu, Z. , Zhou, J. , & Chen, Y. (2018). Tanshinol inhibits the growth, migration and invasion of hepatocellular carcinoma cells via regulating the PI3K‐AKT signaling pathway. OncoTargets and Therapy, 12, 87–99. 30588033 10.2147/OTT.S185997 PMC6304085

